# Chemerin is elevated in multiple myeloma patients and is expressed by stromal cells and pre-adipocytes

**DOI:** 10.1186/s40364-018-0134-y

**Published:** 2018-06-14

**Authors:** Marita Westhrin, Siv Helen Moen, Ida Bruun Kristensen, Glenn Buene, Anne Kærsgaard Mylin, Ingemar Turesson, Niels Abildgaard, Anders Waage, Therese Standal

**Affiliations:** 1 Department of Clinical and Molecular Medicine, Norwegian University of Science and Technology, Trondheim, Norway; 2 Centre of Molecular Inflammation Research, Norwegian University of Science and Technology, Trondheim, Norway; 3 Department of Hematology, Odense University Hospital, Odense, Denmark; 4 Department of Hematology, Rigshospitalet, University of Copenhagen, Copenhagen, Denmark; 5 Department of Hematology, Skane University Hospital, Malmo, Sweden; 6 Department of Hematology, St. Olav’s University Hospital, Trondheim, Norway

**Keywords:** Chemerin, Multiple myeloma, Adipocyte, Stromal cell

## Abstract

Chemerin is a recently discovered adipokine shown to be involved in both inflammatory and metabolic processes. Here, we demonstrate that chemerin serum levels are elevated in patients with multiple myeloma and that it increases with disease progression. We found that chemerin is expressed by stromal cells and preadipocytes, whereas its receptor CCRL2 is expressed by primary myeloma cells, suggesting a paracrine signaling loop between bone marrow stromal cells/adipocytes and myeloma cells. This is the first study exploring chemerin and its receptors in multiple myeloma.

**Electronic supplementary material:**

The online version of this article (10.1186/s40364-018-0134-y) contains supplementary material, which is available to authorized users.

To the editor,

Multiple myeloma is a hematological malignancy caused by clonal proliferation of malignant plasma cells in the bone marrow. Although novel therapies have increased survival of multiple myeloma patients, it remains an incurable disease. Myeloma cells depend on the microenvironment for adhesion, survival and drug resistance. Targeting the microenvironment is vital for novel therapy strategies [1].

Bone marrow adipocytes have emerged as important players in cancer development [2]. They serve as energy reservoirs, secrete fatty acids and adipokines and take part in regulating bone homeostasis. Since myeloma is a disease of the elderly, and adiposity increases with age, the role of adipocytes in myeloma are currently gaining interest. Adiposity has already been shown to increase the risk of myeloma in several studies [3, 4]. Further, it has been shown that adipocytes may affect proliferation, apoptosis and migration of myeloma cells as well as protecting them during chemotherapy [5, 6].

Chemerin (also known as TIG2 and RARRES2) was initially identified as an adipokine involved in adipogenesis and adipocyte metabolism [7]. Chemerin is a modulator of inflammation and it has been suggested as a tumor marker in several cancers [8].

Due to the emerging important role of adipocytes in myeloma, and the role of chemerin in cancers in particular, we measured chemerin serum levels in patients and age— and gender matched healthy volunteers. The patient serum samples were collected at diagnosis during a randomized phase 3 clinical trial, which compared the effect of two different doses of pamidronate on bone in the years 2001–2005 [9]. Out of the original 504 patients, serum samples were available for 122 patients (patient characteristics, Table 1). We found a significant difference between chemerin concentration in patients (mean = 199.2 ng/ml ± 88.2, *n* = 122) and healthy controls (mean = 156.5 ng/ml ± 52.5, *n* = 58) (*p* < 0.001, Mann-Whitney) as shown in Fig. 1a. Chemerin serum levels were significantly higher in patients in ISS stage III (mean = 255.2, ± 106.5, *n* = 28) compared to patients in ISS stage I (mean = 168.2, ± 65.4, *n* = 33) (p < 0.001), and patients in stage II (mean = 189.5, ± 75.8, *n* = 47) (*p* < 0.05, Kruskal Wallis with Dunns post hoc test) as shown in Fig. 1b. As chemerin levels correlated with disease stage we compared overall survival in patients with above (> 192.1 ng/ml) and below median (≤ 192.1) serum chemerin levels. Overall survival did not differ significantly between the two groups (median survival 61.9 months (*n* = 61) for the low chemerin group versus median survival 47.8 months (n = 61) for the high chemerin group, *p* = 0.318 (Mantel-Cox Test). In this patient cohort, chemerin correlated with CRP (spearman *r* = 0.191 *p* < 0.05) and serum β-microglobulin (spearman *r* = 0.332, *p* < 0.001), but not with number of plasma cells (%), the M-component (g/dl), body mass index, s-LDH (ukat/L) or s-calcium (mmol/L). Chemerin serum levels did not associate with bone disease status, or time to SRE. To compare levels of chemerin in the blood and the bone marrow, we analyzed matching blood and bone marrow- plasma samples from 10 myeloma patients (8 from time of diagnosis and 2 from relapse) available at Biobank1®. Chemerin blood plasma levels correlate strongly with chemerin bone marrow plasma levels (spearman *r* = 0.894, *p* < 0.005) as shown in Fig. 1c. There was a tendency to increased amounts of chemerin in the bone marrow plasma compared with levels in blood plasma ((mean = 251.3 ng/ml vs 214.7 ng/ml), *n* = 10).

**Table 1:** Patient characteristics

	Patients (*n* = 122)	Controls (*n* = 58)
Age (years)	62 (37–86)	62 (43–81)
Sex (female)	60 (49%)	31 (53%)
ISS stage		
I	33 (27%)	
II	47 (39%)	
III	28 (23%)	
Unknown	14 (11%)	
M-component		
IgG	75 (61%)	
IgA	29 (24%)	
Light chain	6 (5%)	
Unknown	12 (10%)	
Skeletal affection		
None	12 (10%)	
Limited	49 (40%)	
Osteoporosis	9 (7%)	
Advanced	48 (39%)	
Unknown	4 (3%)

**Fig. 1:**
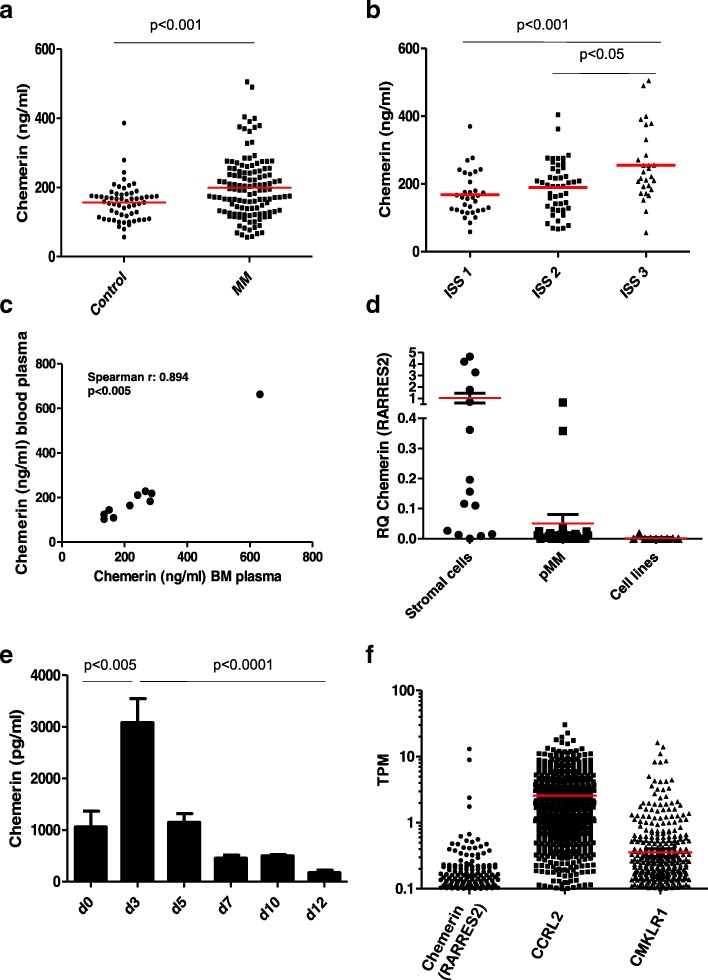
**a** Serum levels of chemerin measured by ELISA (R&D Duoset) in 122 patients, and 58 healthy age and gender-matched controls. **b** Chemerin levels based on ISS stage. **c** Blood- and bone marrow plasma levels of chemerin in myeloma patients obtained from our biobank (*n* = 10). **d** PCR analysis of chemerin (RARRES2) expression by myeloma-derived stromal cells (*n* = 15), primary myeloma cells (*n* = 24) and myeloma cell lines (*n* = 9). **e** Healthy bone marrow-derived mesenchymal stromal cell donors differentiated towards adipocytes (*n* = 3 donors) according to the manufacturer’s instructions. Conditioned cell culture media were harvested at every medium change. Chemerin levels in the conditioned media were measured by ELISA (R&D Duoset). Error bars = SEM **f** Expression of chemerin (RARRES2), CCRL2 and CMKLR1 in malignant plasma cells from 673 myeloma patients available in the CoMMpass database, release IA9. Red bars indicate mean in **a**, **b**, **d** and **f**

To examine which cells may be producers of chemerin in the bone marrow we examined gene expression by qPCR in stromal cells isolated from myeloma patients (*n* = 15), primary myeloma cells (*n* = 24) and myeloma cell lines (*n* = 9). We found that chemerin was significantly higher expressed in stromal cells compared with primary myeloma cells, and that myeloma cell lines expressed lower levels than primary myeloma cells, Fig. 1d. Since chemerin was initially identified as an adipokine we further measured chemerin protein levels in the supernatants from bone marrow-derived mesenchymal stromal cells (*n* = 3) differentiated towards adipocytes using the hMSC Adipogenic Differentiation BulletKit™ Medium (Lonza). Interestingly, pre-adipocytes produce and secrete chemerin at high levels, as shown in Fig. 1e. The increase in chemerin protein levels corresponded with an increase in chemerin mRNA levels (not shown).

Chemerin mediates effect through its receptors CMKLR1 and GPR1, but it can also bind to CCRL2, which is a non-signaling receptor [10]. To examine receptor expression for chemerin in primary myeloma cells, we utilized the CoMMpass data series, which contains RNA-sequencing data from 673 newly diagnosed myeloma patients. We found that CCRL2 mRNA was expressed in most of the patients while CMKLR1 mRNA was expressed in a fraction of the patients. In line with qPCR results in Fig. 1c chemerin (RARRES2) was not expressed by primary myeloma cells, Fig. 1f. Interestingly, primary myeloma cells expressed more CCRL2 and CMKLR1 mRNA compared with multiple myeloma cell lines (Additional file 1: Figure S1).

In conclusion, chemerin levels in serum are elevated in patients with multiple myeloma compared to healthy controls. Chemerin serum levels correlate with disease stage. Stromal cells obtained from myeloma patients and pre-adipocytes produce chemerin, whereas the receptors CCRL2 and CMKLR1 are expressed by myeloma cells. Thus, we propose that chemerin may mediate a paracrine signaling loop between stromal cells/adipocytes and myeloma cells.

## Additional file

Additional file 1: **Figure S1.** Expression of CCRL2 and CMKLR1 in primary myeloma cells (pMM, *n* = 24) and cell lines (*n* = 9) analyzed by qPCR. GAPDH was used as an endogenous control. (DOCX 51 kb)

## Abbreviations

CCRL2: C-C chemokine receptor-like 2
CMKLR1: Chemerin Chemokine-Like Receptor 1
GPR1: G Protein-Coupled Receptor 1
hMSC: Human mesenchymal stromal cell
ISS: International staging system
RARRES2: Retinoic Acid Receptor Responder 2
SRE: Skeletal related event
