# Supplementary material for: Chemerin is elevated in multiple myeloma patients and is expressed by stromal cells and pre-adipocytes
Source: Biomark Res. 2018 Jun 14;6:21. doi: 10.1186/s40364-018-0134-y (PMC6001014; doi:10.1186/s40364-018-0134-y)
Supplement: Supplementary file 1 — Figure S1. Expression of CCRL2 and CMKLR1 in primary myeloma cells (pMM, n = 24) and cell lines (n = 9) analyzed by qPCR. GAPDH was used as an endogenous control. (DOCX 51 kb) [file 40364_2018_134_MOESM1_ESM.docx]

**Additional file 1: Figure S1**


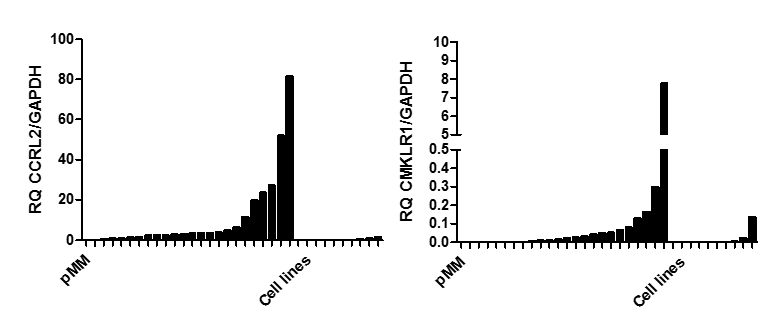


**Figure S1.** Expression of CCRL2 and CMKLR1 in primary myeloma cells (pMM, n=24) and cell lines (n=9) analyzed by qPCR. GAPDH was used as an endogenous control.
